# Biases in Viral Metagenomics-Based Detection, Cataloguing and Quantification of Bacteriophage Genomes in Human Faeces, a Review

**DOI:** 10.3390/microorganisms9030524

**Published:** 2021-03-04

**Authors:** Julie Callanan, Stephen R. Stockdale, Andrey Shkoporov, Lorraine A. Draper, R. Paul Ross, Colin Hill

**Affiliations:** APC Microbiome Ireland and School of Microbiology, University College Cork, T12 YT20 Cork, Ireland; julie.callanan@umail.ucc.ie (J.C.); stephen.stockdale@ucc.ie (S.R.S.); andrey.shkoporov@ucc.ie (A.S.); l.draper@ucc.ie (L.A.D.); p.ross@ucc.ie (R.P.R.)

**Keywords:** bacteriophage, viral metagenomics, phageome, viral extraction

## Abstract

The human gut is colonised by a vast array of microbes that include bacteria, viruses, fungi, and archaea. While interest in these microbial entities has largely focused on the bacterial constituents, recently the viral component has attracted more attention. Metagenomic advances, compared to classical isolation procedures, have greatly enhanced our understanding of the composition, diversity, and function of viruses in the human microbiome (virome). We highlight that viral extraction methodologies are crucial in terms of identifying and characterising communities of viruses infecting eukaryotes and bacteria. Different viral extraction protocols, including those used in some of the most significant human virome publications to date, have introduced biases affecting their a overall conclusions. It is important that protocol variations should be clearly highlighted across studies, with the ultimate goal of identifying and acknowledging biases associated with different protocols and, perhaps, the generation of an unbiased and standardised method for examining this portion of the human microbiome.

## 1. Introduction

The estimated number of bacteriophages (phages) within the human gut has been recently calculated as approximately 10^10^ per gram of faeces [1]. The genetic material encapsulated within these phages is either DNA or RNA, which in turn can be double-stranded (ds-) or single-stranded (ss-). The single-stranded variants can exist in two different forms depending on their orientation and polarity: positive-sense or negative-sense. No negative-sense ssRNA phages have been identified to date.

It has been over a decade since the first attempts to conduct metagenomic analyses of gut viral communities [2]. Many studies in this area have deposited their data in public databases. We are particularly interested in the phages that may influence the composition, turnover and functionality of bacterial communities [3,4]. The number of studies focusing on this phage population, termed the ‘phageome’, has increased in recent decades [1]. This surge in phageome research has been made possible by advances in contemporary sequencing technologies and specialised virome sequencing data analysis tools including VirSorter and Demovir [5,6].

There have been efforts to create standardised protocols to study the faecal phageome through metagenomic analyses that allow for reliable comparisons between studies from different groups [7,8]. One such effort was that of Conceição-Neto et al. (2015) in which they proposed the ‘Novel enrichment technique of VIRomes’ (NetoVIR) protocol [9]. This method was designed using mock viral and bacterial communities which included both +ssRNA and dsRNA viruses, which were not phages, but does suggest an approach to optimise their recovery. Nevertheless, the search for common protocols enabling cross-study comparison should not discourage researchers from developing novel techniques to capture new phages. Most newly identified phage sequences do not have known counterparts in viral databases, and these unknown sequences are often collectively referred to as the ‘viral dark matter’ [1,10,11]. It has been revealed that the viral dark matter can account for 60–95% of the genomes identified [11,12].

The majority of newly discovered phages may be novel because (i) their bacterial hosts are recalcitrant to isolation and cultivation, (ii) they exhibit unusual or previously undescribed lifecycles which may prevent them from being detected using the typical plaque-dependent methods, or (iii) there is a strong likelihood that the methods used may not have been suitable and more effort is required to capture all types of phage [13,14,15]. Considering that many bacteria are yet to be grown in a laboratory, culture-based methods are limited in their efficacy for isolating new phage-host pairs [16]. Even when the host is culturable, the phage may not plaque as it may not infect until the host has reached a specific growth phase [17], the plaques may be very difficult to see if the phage diffuse poorly in agar, or it may be lysogenic or practice pseudolysogeny (the delayed development of a phage in the host cell) [18]. It could be a result of differences in the physiology of a bacterium in a laboratory environment compared to growth in its natural environment [19]. In an effort to bring order to these novel sequences, collaborative efforts are required to link both metagenomic analyses and culture-based investigations. One example is the successful isolation of the first crAss-like phage. CrAss-like phages are viruses with relatively large genomes (~100 kb) that were originally found in metagenomic studies and predicted to infect bacteria in the order Bacteroidales. Collectively, this phage family group is the most abundant human gut-associated viral clade, identified in >50% of people, and representing up to 90% of all sequencing reads in some human gut viromes [20,21,22]. Through a combination of bioinformatic-based discovery and subsequent laboratory-based experiments, the first representative of this family of phages was isolated and propagated on its *Bacteroides intestinalis* host [23].

The lack of a single phylogenetic marker in virology (equivalent to the 16S rRNA or the *chaperonin-60* (*cpn60*) gene in bacteria) further complicates our ability to properly assign taxonomic ranks to this ‘viral dark matter’. A recent publication has described Minimum Information about an Uncultivated Virus Genome (MIUViG) standards in an attempt to overcome this difficulty [24]. These include virus origin, genome annotation and quality, taxonomic classification, and a collection of other mandatory and optional metadata. Community-wide compliance with these standards will allow for more effective evaluation of the global virosphere and more robust comparisons between studies.

**Figure 1:**
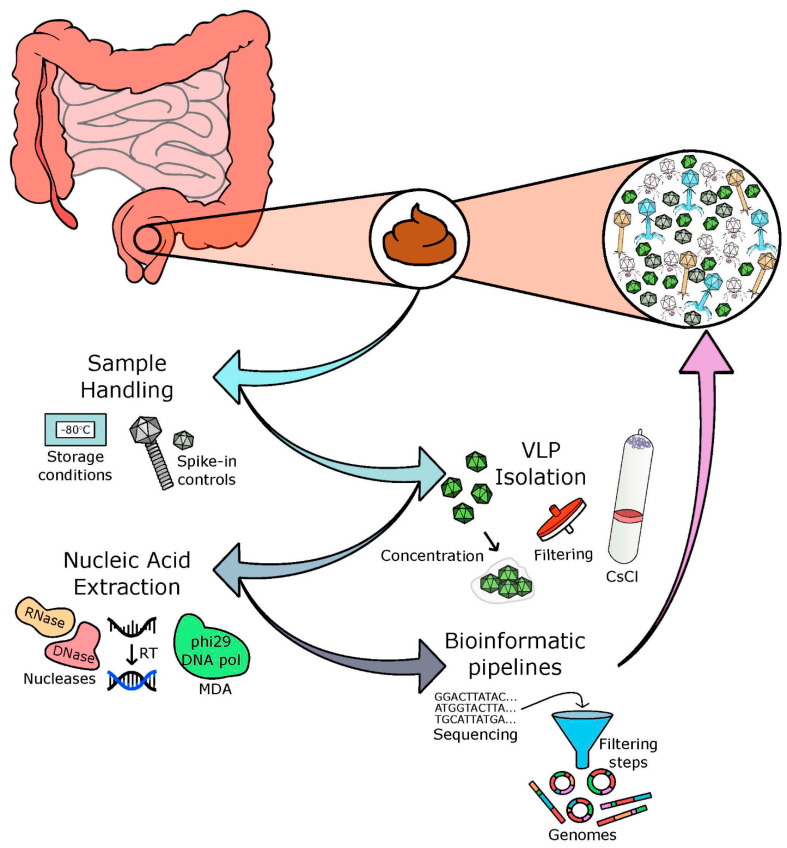
Basis of a viral/phage isolation protocol. Faecal samples are often used as a proxy for the human gut virome. Through four main processes, the viral and phage communities of the human gut are analysed: (**i**) acquisition and storage of samples, (**ii**) concentration of viral particles, (**iii**) extraction of pure nucleic acids with the elimination of free nucleic acids, and (**iv**) successful sequencing and bioinformatic analysis of these nucleic acids.

The characterisation and quantification of nucleic acids of uncultured viruses isolated from different biomes is dependent on many factors, including concentration, purification, extraction, and sequencing techniques. There is no ideal ‘capture-all’ protocol [25] but care and consideration is crucial in relation to the choices made at each stage of the protocol. There are four main processes involved in the development of a phage sequencing protocol, including: (i) acquisition and storage of the sample, (ii) separation of viral particles, (iii) the extraction of pure nucleic acids with the elimination of free nucleic acids and contaminating cells, and (iv) successful sequencing and bioinformatic analysis of these nucleic acids (as depicted in Figure 1).

Despite rapid advances in high-throughput sequencing technologies, few studies detect RNA viruses in human and animal faecal samples, even when a reverse transcriptase step is included. This may be due to low RNA viral loads, destabilisation of the viral particle, reliance on physical virion characteristics, or as a direct result of the nucleic acid extraction method used in the study [7]. It has been suggested that RNA viruses form an important part of the total gut virome but most studies to date have concluded that members of RNA phage families are only a minor component [2,26,27,28]. However, while RNA phages were rarely detected in environmental metagenomics, recent studies have reported logarithmic increases in the total number of known single-stranded RNA phages from these sources [29,30,31]. Therefore, gut RNA phages may be underestimated and without comprehensive studies targeting these elusive phages, the biological significance of RNA phages may remain largely overlooked.

There is an unavoidable loss of some virions at almost every step of the protocol. Viral particles can become adsorbed to larger molecules such as food particles, immobilised on filters or damaged by nucleases. Certain viruses such as giant viruses that can reach 750 nm in size may also be excluded in some filtering protocols, while filamentous viruses such as *Lipothrixviridae* can often reach over 2 μm in length [32,33]. Both these virus types will not pass through the majority of filtering processes. Conceição-Neto et al. (2015) also highlighted that the use of small filter pores, coupled with strict centrifugation conditions, may lead to the exclusion of these larger viruses from virome analyses [9]. In the development of an optimised viral isolation protocol, it can be difficult to balance increasing contamination risk with larger particles and smaller bacterial cells for the possible reward of incorporating these viral types.

Here we examined the biases associated with the key isolation steps used in published virome studies. This was performed by surveying studies over the past decade that have started with a faecal sample and used different methods to examine the human gut virome, with particular attention on phageome composition and recordings of RNA viruses. It is hoped that by addressing any shortcomings of current methods and identifying crucial procedures in retaining the true viral diversity of the human gut, a standard or reference protocol could be developed that would be reproducible and comparable across research studies focusing on reducing method biases and including RNA phages.

## 2. Sample Handling

The quantity of sample required is dependent on the efficiency at which viruses can be isolated. In this review we focus on studies using faecal samples. Faecal samples have been extensively used as a starting material in order to study the complex virome and phageome associated with the human gut. Faeces are also widely used as they offer a more practical and non-invasive means to access novel phages from the gut. This is compared to other sampling sites of the gastrointestinal tract (GIT) which are ethically and practically more difficult to acquire, such as biopsies from the human GIT mucosa. Nearly two decades have passed since the initial analysis of the composition and population structure of the uncultured viral community from human faeces [2]. Breitbart et al. noted that the majority of sequences were unrelated to previously known sequences, and the most recognisable were those belonging to *Siphoviridae*, a family of dsDNA phage belonging to the order *Caudovirales*. Since then, treatments, techniques, and protocols associated with extracting the viral and phage fractions from human faeces have improved.

While storage temperatures of samples could potentially contribute to virome composition, Shkoporov et al., demonstrated that repeated freeze–thaw and alternative storage temperature (4 °C vs. room temperature) only had a mild effect on the dsDNA composition of the virome [7]. In the same study researchers assessed the effect of freeze–thaw cycles on elusive gut phages and found that this treatment affected the bacterial components more so than the phage population. It is suggested that where possible it is best to avoid repeated freeze–thaw cycles of the sample and the sample should be frozen as soon as possible [34].

The choice of buffer also requires careful consideration. The majority of virome studies use either a buffer composed of a mixture of sodium chloride and magnesium sulphate (SM buffer) or phosphate-buffered saline (PBS) buffer as a means to resuspend the faecal sample and release viral particles. There have been suggestions that SM is the preferred option due to the potential of the different ions to inactivate the phage, for example Adams observed that phosphate ions inactivate Enterobacteria phage T5 [35].

The inclusion of a spiked-in exogenous phage standard, such as the lactococcal phage Q33 or +ssRNA phage Qβ, enables a semi-quantitative analysis of individual members of the virome following metagenomic sequencing by analysing the percentage of reads aligning to these spiked-in genomes [7]. It should be noted that no reads aligning to the spiked-in +ssRNA phage were detected in downstream analyses of that study. In 2019, d’Humières et al. examined the effect that four different methods, varying only in the phage concentration step, had on the overall phageome composition from the same faecal material [8]. They noted that the initial mechanical agitation is essential to dissolve the phage particles in the faeces when homogenised in PBS. Therefore, protocols should note at which point in the preliminary stage of the protocol the spiked-in controls are added.

## 3. VLP Isolation

The purification of virus-like particles (VLPs) is one of the most critical steps in the quantitative and qualitative metagenomic analyses of the total viral population. Ideally one would want to reduce the number of contaminating bacterial sequences present in the VLP fraction. Bacterial genomic DNA/RNA detected in downstream analysis could be due to contamination or may have been packaged within phage particles as a result of generalised transduction, specialised transduction, or incorporation in Gene Transfer Agents (GTAs) [36,37]. In the majority of virome and phageome work, centrifugation and filtering of faecal supernatants are used to eliminate debris and remove bacteria.

Many studies report the level of contamination associated with their samples, both of bacterial and human origin. One such example is a study by Norman et al. (2015) where analysis of their VLP sequences revealed a low level of contamination with human sequences (0–4%) and they acknowledged that there was also possible contamination with bacterial sequences that was confounded by the presence of integrated prophages in full genome sequences of bacteria [38]. This highlights how essential it is to identify contaminating particles from the sample following both physical and bioinformatic filtering in downstream processes. Some groups have adopted a novel approach of identifying contaminating bacterial sequences which align to *cpn60*, a highly conserved house-keeping gene. As the *cpn60* gene occurs once per genome it offers an alternative bacterial taxonomic marker to the traditional 16S rRNA and also gives finer taxonomic discrimination between bacteria [1,7,39,40]. The analysis of the *cpn60* gene overcomes concerns that the 16S rRNA gives disproportionally high levels of bacterial contamination due to the rRNA being purified in workflows in the form of ribosomes being co-isolated along with viral particles.

It had been previously demonstrated under microscopic examination that 0.22 µm filters reduced the number of viral particles from faecal samples by almost half [41]. Indeed, in the previously mentioned d’Humières study, it was also found that the filter size is crucial in the early stages of phageome studies [8]. Their results showed that filtration should be done using 0.45 µm and 0.2 µm filters and not just 0.22 µm as the combination of filter sizes allows the faecal lysate purified of larger contaminants prior to selecting for the VLP portion.

Polyethylene glycol (PEG) precipitation is often used in protocols to concentrate the sample prior to sample purification or nucleic acid extraction. In the study by d’Humières et al., they deemed the method including PEG to be the best of those examined across a range of faecal samples in order to assure reproducibility and sequencing depth [8]. They also discussed the efficacy of the method including PEG to concentrate phages from faecal filtrate, suggesting that despite the fact it requires an overnight incubation step, it would be a beneficial reagent to include in phage isolation protocols. This requirement for chloroform in the PEG-removal step, and at different points in nucleic acid extraction protocols, has repercussions as chloroform degrades and destroys the phospholipid membrane of some enveloped viruses, such as dsRNA phages of the family *Cystoviridae*, potentially leading to dramatic under-representation of such viral groups.

Tangential-flow filtration (TFF) can also be used to concentrate viral particles from samples. Thurber et al. (2009) discussed the advantages and disadvantages associated with this method and ultimately decided to exclude it from their final protocol [25]. The main flaw associated with TFF is that in order to maximise viral recovery, approximately two volumes of the filtrate is recirculated which results in a dilute final retentate. This approach is better suited to non-faecal samples and is routinely used to study aquatic environments. Similarly, zinc chloride and ammonium acetate precipitation protocols to concentrate phages are more suited to non-faecal samples [42,43].

Another method commonly used for viral particle purification is caesium chloride (CsCl) density centrifugation which is based on physical properties of the virion [44]. The factors associated with CsCl purification include the speed of the centrifuge, the solvent used for resuspension of faeces and the number of gradient layers examined and are dependent on the virus buoyant density. Researchers should always make the gradients from the same buffer present in the samples and filter-purify the gradients to reduce the levels of contaminating viruses in the final fractions. It is crucial not to unsettle the borders of the layers and to fill the column tube completely prior to centrifugation. Phages are concentrated in a multi-layer gradient where they are localised at different densities and subsequently removed using a sterile needle. The CsCl gradient selection and type are crucial factors to consider prior to excision of bands as it may inadvertently exclude RNA phages and skew the outcome in favour of DNA phages. In efforts to recover RNA phages from this method, as well as the DNA phages, multiple different bands should be excised and examined. A summary table of this information is available by Fauquet et al., in which they note the densities and sensitivities associated with RNA phages [44]. It should be noted that this may introduce biases to the resulting population as phages and other viruses outside this range may be excluded based on the selected densities.

In the d’Humières study, it was found that the method including a CsCl step gave the lowest bacterial contamination and largest contigs but also had the lowest phage diversity, was very time consuming, and showed poor reproducibility [8]. These key points agreed with the findings from another study by Kleiner et al. where they examined the effects of different extraction methods on an artificial intestinal microbiota sample [45]. Some studies specifically noted that this optional step is excluded for being too labour intensive and inappropriate for high-throughput studies.

## 4. Nucleic Acid Extraction and Library Preparation

Once the VLP fraction has been separated from the faecal material, there are a series of necessary steps to allow for the isolation of the nucleic acids. This is essential to yield nucleic acids of sufficient purity and concentration for downstream library preparation and sequencing [46]. Although the vast majority of virome studies have solely focused on the DNA portion, metagenomes of RNA viruses have also been generated. It is crucial that RNase-free and viral-free reagents be used in the isolation of the RNA. These studies rely on creating sufficient quantities of cDNA via reverse transcription of the viral RNA.

There are some studies that have combined the use of the phenol/chloroform protocol for bacterial nucleic acid extraction and the formamide/cetyltrimethylammonium bromide (CTAB) method which was traditionally used for the extraction of viral DNA. The main advantages of these methods compared to commercially available kits are the decreased associated costs and the absence of a carrier RNA. This additional carrier RNA functions as a means to enhance the recovery of DNA/RNA by preventing the target nucleic acids in low yield samples from being irretrievably bound and increasing the success of downstream PCR processes [47]. This carrier RNA can often contaminate samples and requires the addition of an additional RNase step to remove it.

A recent review by Garmaeva et al., (2019) discussed the impact of nucleic acid extraction protocols on the observed composition of the human gut virome and the apparent dominance of DNA viruses, particularly dsDNA phages, from faecal samples used in different studies [7,9,26,27,45,48]. They highlighted the fact that our current understanding of the human gut virome composition may underestimate the abundance and importance of the RNA viral portion. The inclusion of an RNase step in the treatment of the faecal sample is commonly used to remove free non-viral contaminating RNA [7,45]. However, recent work has demonstrated that the addition of RNase negatively affects the RNA-fraction of the virome [49]. Some RNA viruses also contain portions of the RNA as a component of their nucleocapsid structure while others have loose capsid structures which make the virus susceptible to RNase degradation. This structure may be destroyed by the addition of RNase, as demonstrated by Acheson and Tamm with their findings that Semliki Forest virus nucleocapsid disintegrated following RNase treatment [50]. Despite the risk of increased contamination levels with rRNA or cellular mRNA, it may be wise to restrict the use of RNases in order to capture the true RNA viral diversity and instead implement stricter filtering steps in the bioinformatic quality control workflows. The inclusion of a DNase step is still widely accepted as an essential step in the removal of contaminating free DNA fragments from the sample.

With the low yield of phage nucleic acids from extraction methods and the high amount of DNA required for library preparation kits, many researchers have had to rely on multiple displacement amplification (MDA) prior to sequencing. It has been widely shown that this amplification protocol preferentially amplifies small and circular ssDNA [8,51,52]. MDA is dependent on the high processivity of the phi29 DNA polymerase, an enzyme with strand-displacement activity which allows for amplification of genomic DNA using random primers with a single denaturation step [53]. This type of practice is referred to as a whole genome amplification (WGA), a robust method to amplify the entire genome of limited extracted nucleic acid samples. There is an ongoing controversy regarding the use of this method in phageome studies as it appears to introduce bias affecting the relative frequency of dsDNA phages of the *Caudovirales* order (including *Siphoviridae*, *Myoviridae* and *Podoviridae* families) and +ssDNA phages of the *Microviridae* family in the healthy human gut [7,26,27,54,55]. Several of these studies have included an MDA step before sequencing [7,27,38,54,56], whereas some studies use kits, such as the Nextera XT DNA Library Prep kit, without the MDA step to exclude ssDNA phages [8,57]. Another issue associated with the inclusion of an MDA step is its inability to capture RNA viruses [58]. A study in 2010 by Reyes et al. examined the effects that MDA had on their samples by comparing an unamplified sample to an MDA/WGA processed sample [27]. They determined that 98.4% of unamplified sequences were present in the WGA, while 91.96% of the WGA were reciprocally found in the unamplified sample. These discrepancies may be due to the preferential bias in the amplification of small ssDNA viruses, as also corroborated by other studies [38,57]. Recent work by Gregory et al., examining age-dependent patterns of the human gut virome using pre-existing datasets, found that 96% of studies were MDA treated [59] It has been suggested that MDA should be avoided where possible, as it can result in less diversity and less reproducible outputs. Some studies have completely avoided this step to evade potential amplification biases [60]. To overcome this bias, improvements in the library preparation protocols are required which will also lead to metagenomic studies of the human gut phageome becoming more representative of the true composition [57].

A study by Lim et al. in 2015 utilised both MDA and sequence-independent amplification (SIA) of the DNA and RNA, although they noted that the SIA method is less sensitive for DNA viruses [58]. It was used to balance the MDA as it can capture RNA viruses, although it is generally less sensitive in terms of DNA virus representation. The SIA method involved in this study incorporated base-balanced specific 16nt sequence upstream of a random 15-mer for random priming. It is based on the flanking of unknown sequences with known sequences to enable PCR amplification [61]. Nonetheless, there were no RNA phages detected in the SIA-generated data and the authors focused on the MDA-generated data to make their results comparable with other virome and phageome studies [58]. They identified picobirnaviruses that were previously classified as eukaryotic viruses but have more recently have been suggested to be dsRNA phages as they contain conserved prokaryotic ribosomal binding sites [62]. Lim et al., also identified eukaryotic RNA viruses including *Caliciviridae*, *Picornaviridae* and *Astroviridae* which are +ssRNA viruses (with non-segmented genomes) from the infant faecal samples. This is consistent with results from other PCR-based studies [63]. In a 2015 study by Norman et al., there was also quite a high relative abundance of dsRNA viruses, retro-transcribing viruses (these contain ssRNA) and unclassified phages, some of which may represent undefined RNA phages [38]. These viral and phage contigs may be detected as a result of alterations made to a previous protocol including the removal of an RNase step.

**Figure 2:**
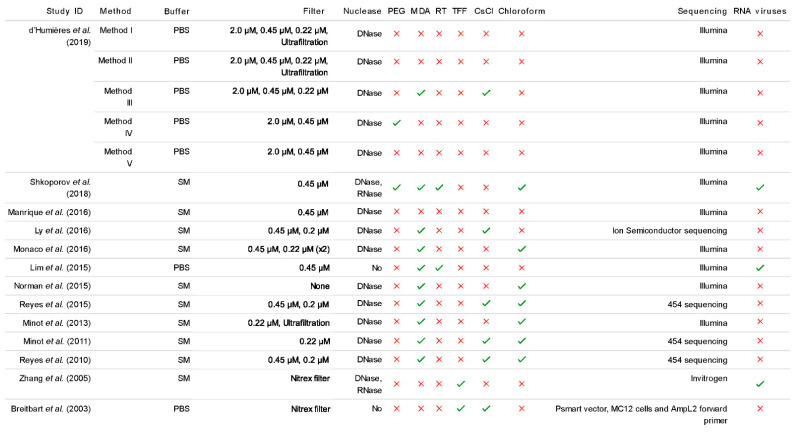
Summarised extraction method comparisons from a selection of recent human gut virome papers. All studies included in this review analysed the composition of the human gut virome based on faeces as the starting material [2,7,8,26,27,28,38,54,58,60,64,65,66]. The different sample handling methods, procedure for extracting the VLP fraction and sequencing technology are examined. In the 2019 study by d’Humières et al., method I includes ultrafiltration whereas method II involves both ultrafiltration and ultracentrifugation. (See Supplementary Material for data).

Some studies have attempted to incorporate the typical protocols for RNA phage isolation from the VLP fraction, such as reverse transcription (RT), but not all record recovering RNA phage (Figure 2). The resulting DNA, known as complementary DNA (cDNA) can then be used as a template for PCR reactions. There may also be a MDA step coupled with the RT step in order to convert a sample from an ssDNA/ssRNA heteroduplex into dsDNA. This is crucial for the enzyme associated with the library preparation kit to work efficiently as they are selective to the nucleic acid sample, in the case of the transposase in the Nextera XT Library Preparation Kit which requires dsDNA input.

Shkoporov et al. acknowledged the fact most studies on the human phageome have neglected to study the RNA fraction, with a few notable exceptions and in their study, attempted to incorporate the RNA viral consortium by including a RT step [7]. Interestingly, the authors highlight the fact the protocol may have failed to quantitatively recover the small +ssRNA phage that was deliberately spiked in the sample.

In a preliminary small-scale RNA-focused study, Zhang et al. performed a metagenomic study of the uncultured RNA viruses residing in the human gut and indicated that the majority (>95%) of these were plant viruses, the most abundant found to be pepper mild mottle virus (PMMV) [28]. They did detect a large amount of hits for the animal virus *Picobirnavirus*, which as previously mentioned may in fact be a dsRNA phage [62]. However, in this study the method of extraction was based on TFF and both DNase and RNase were added which may affect the sensitivity of this method to find other RNA viruses and phages. This finding led to a debate of to what extent RNA viruses and phages inhabit the human gut and if there is any significance in attempting to capture these entities. Several studies have been published that demonstrate the abundance of RNA phages in non-faecal metatranscriptomic samples such as activated sludge, seawater, insect, and avian samples [29,30,31,67].

Next-generation sequencing (NGS) includes approaches that are non-Sanger method-based high-throughput DNA or cDNA sequencing methods which, in brief, operate by the initial fragmentation of the DNA/RNA into shorter fragments, the ligation of terminal adapter sequences, amplification and sequencing of these libraries (based on one out of several available chemical or physical principles) and, finally, an attempt to assemble these short sequences into larger contigs, or even complete genomes. Following the advances made in NGS in recent years, cost-effective and rapid sequencing platforms such as Illumina HiSeq, and third generation long-read sequencing platforms such as Oxford Nanopore, have become more accessible. Prior to sequencing, NGS libraries are prepared from the isolated viral nucleic acids which have been fragmented to particular lengths to comply with the specific sequencing platform chosen. Subsequently, there may be a series of preparation steps in which special adapters are added to allow single entities to be identified when the samples are pooled for sequencing runs. These adapters also provide priming sites for amplification after ligation, priming sites during isothermal bridge amplification inside flow cells and at the sequencing step. Enhanced library preparation, such as Nextera XT and Accel-NGS^®^ 1S DNA library kits, have allowed for quicker and more efficient sequencing from limited amounts of the DNA/cDNA starting material. Prior to library preparation, the proper type and amount input material is essential, for example Nextera XT requires dsDNA input so any sample with a potential DNA/RNA heteroduplex will require additional treatment. Once the library has been prepared, sequencing using a NGS-specific platform, such as Illumina MiSeq or HiSeq, is performed and the results are then analysed.

The sequencing depth of the samples is also something that needs to be considered prior to selecting the sequencing platform as different platforms offer varying length (bp), throughput and number of reads and can often dramatically range in cost per gigabyte (Gb) [68]. For example, considering short-read NGS using Illumina with long-read NGS by Oxford Nanopore (MinION), there are notable difference in length (25–300 bp vs. up to 200 kb, respectively), throughput (2–900 Gb vs. up to 1.5 Gb, respectively) and number of reads (10 M–4 B vs. >100 k, respectively) and these factors need to be addressed in order to select the best platform for the specific study. The majority of studies examined in this review used Illumina (usually either HiSeq or MiSeq) as the preferred sequencing platform.

A study by Castro-Mejía et al. attempted to optimise the extraction and purification of phages from human faecal samples prior to metagenomic analysis [69]. They separated the process into two parts; the pre-processing, which included the spiking of three phages, and the purification which included PEG, TFF and an adapted method from the literature. Despite the fact these protocols were found to be highly efficient in the purification of DNA phages prior to high-throughput sequencing for phage-metavirome studies, their efficacy at recovering RNA viruses from such samples is yet to be tested.

## 5. Bioinformatic Pipelines

In order to validate the various isolation protocols and combinations, a specific and robust bioinformatic pipeline is essential. Following on from the VLP isolation, extraction and sequencing of the nucleic acids, the resulting viral sequences are analysed in order to identify and characterise the viral contigs. A recent literature review by Nooij et al. examined 49 bioinformatic workflows for viral metagenomics which led to the creation of two decision trees which can be applied to a variety of viral analyses [70]. The vast amount of data derived from NGS has resulted in challenges with the quality analysis and the processing of the sequences. To help circumvent the demanding nature of some of these processes and to make metagenomic analyses more accessible, online tools and resources have been developed. There are virome-specific programs such as Viral MetaGenome Annotation Pipeline (VMGAP) [71], Viral Informatics Resource for Metagenomic Exploration (VIROME) [72], and Metavir 2 [73]. The aforementioned tools are dependent on reference databases as they operate on a similarity-based system, but there are several similarity-independent resources that have also been developed such as PHAge Communities from Contig Spectrum (PHACCS) [74]. This enables the user to bypass issues that may arise due to a lack of sequence similarity in databases [75].

The complete collection of bioinformatic resources has been reviewed and new tools are constantly emerging in an attempt to better analyse the sequences [76]. These include VirSorter [5], DemoVir [6], DeepVirFinder [77], Detection & Analysis of viral and Microbial Infectious Agents by NGS (DAMIAN) [78] and numerous others. There are also studies that use tailor-made pipelines, such as that by Monaco et al. who used a bioinformatics pipeline, VirusSeeker, to analyse their viral sequences [65].

**Figure 3:**
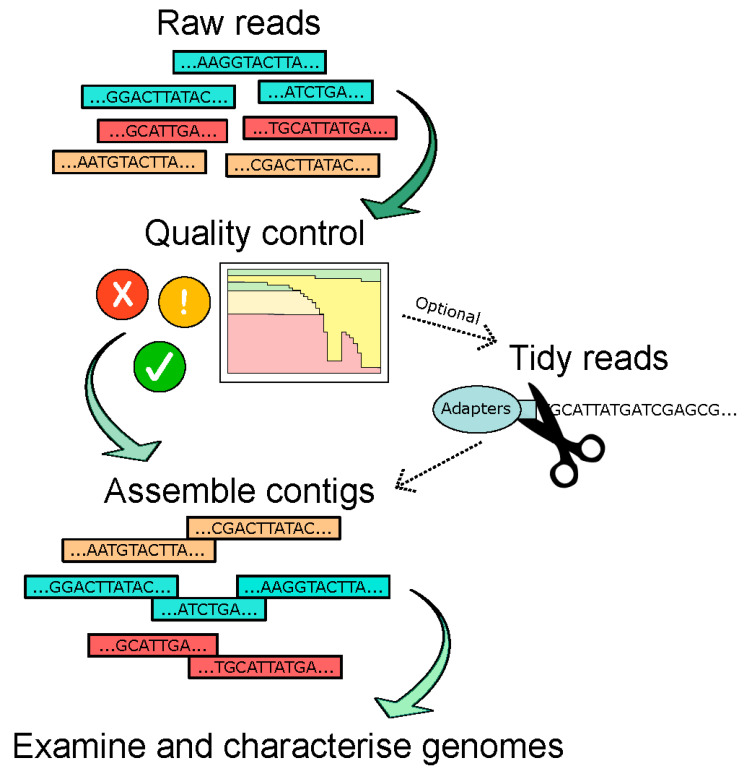
Overview of genome assembly as part of the bioinformatic pipeline used for virome/phageome analyses.

The choice of assembly software used in different studies may offer a source of differentiation in studies as it has been recently shown that this has a critical impact on the recovery of the viral contigs (see Figure 3 for basic bioinformatic pipeline) [14]. Certain criteria, such as genome circularity, contig length, presence of particular phage proteins, and percentage identity to known viruses, are also applied to further filter the viral contigs. However, it should be noted that many of these filters could remove the RNA viruses—for instance, the particular step detecting circular genomes would exclude all known RNA phages. In a 2015 paper by Reyes et al., where the DNA gut virome of Malawian twins was analysed, circular contigs were used as a criterion and revealed three distinct size ranges for circular contigs: (i) >30 kb (the reported size range for circular dsDNA phages belonging to the *Caudovirales* order); (ii) 6–7 kb (size reported for ssDNA phages in the *Microviridae* family, particularly the *Alpavirinae*); and (iii) 3–4 kb (expected size for ssDNA eukaryotic viruses in the *Anelloviridae* family) [66]. It is also important that assembly statistics are reported in studies to evaluate the quality of the assembly. One such example is the N50 which, in simple terms, denotes the shortest contig used to represent 50% of the assembled genome [79]. Therefore, it is a measure of the quality of assembled genomes and the degree of fragmentation.

Decontamination of samples to remove bacterial and other non-viral sequences can be done by positive or negative selection, i.e., filtration and selection of viral contigs from the total sample or the identification and removal of non-viral sequences, respectively. The compositional profile of the viral sample is often assessed by aligning the assembled reads to a reference database of known viruses using Basic Local Alignment Search Tool (BLAST) or other BLAST-based programs. Alternative sequence analysis methods such as *k*-mer algorithms, such as VirFinder and Libra, can reduce the time required for these analyses [80,81]. It is important to note that these programs can be extremely computationally heavy to work at such speeds. This *k-mer* based method is rare but has been used in some virome studies, including the gut virome study by Norman et al. [38]. These searches are restricted to reference databases like the NCBI Reference Sequence (RefSeq) database that are limited in the level of annotation they offer as they represent only a modest proportion of the total global virome [82]. Other references databases also exist such as Reference Viral Database (RVDB), which includes all viral sequences except for bacterial viruses [83], ViPR database [84], and GenBank, which is a collection of all annotated sequences [85].

There are also custom-built profile hidden Markov model (HMM) databases generated through the collection of conserved viral proteins, e.g., the Prokaryotic Virus Orthologous Groups [pVOGs] database [86]. Another example is the recent publication which utilised a profile-HMM database of conserved +ssRNA phage proteins to expand the number of these entities from tens to thousands [29]. This tool will enable the identification of +ssRNA phages that are somewhat closely related to those already known and more distantly related strains from future studies.

In the MIUViG paper, Roux et al. discuss how the numbers of viral reference databases are being created at extraordinary rates but these are rarely deeply examined for inflated dataset novelty [24]. As the number of virome studies increases year on year, the number and breath of the databases available should increase which will allow for more robust assigning of identity to viral contigs.

## 6. Discussion

Efforts to examine the phageome and overall virome of the human gut through the analysis of the viruses and phages from a faecal sample are reliant on the accuracy and reproducibility of various protocols and subsequent analyses. Shkoporov et al., noted that the available data on the conclusive concentration of the VLP fraction of human faecal samples are repeatedly contradictory and heavily dependent on the extraction and quantification procedures applied [1]. Throughout the various studies there have been notable sources of bias, ranging from the inclusion of MDA treatment to the addition of RNase. By highlighting these biases, efforts to avoid such procedures can be made and alternative techniques can be tested. It is also essential that for all viruses and phages to be equally represented, both the RNA and DNA portions need to be examined in future phage-metavirome studies.

## Data Availability

Not applicable.

## Supplementary Materials

The Supplementary Materials are available online at https://www.mdpi.com/2076-2607/9/3/524/s1. The table and R script used in the analysis and creation of Figure 2 are provided.
